# *Candida albicans* hyphae modulate *Staphylococcus aureus* cell-free supernatant during dual biofilm growth to drive molecular signatures of oral dysplasia

**DOI:** 10.1007/s00430-026-00880-4

**Published:** 2026-06-19

**Authors:** Freddy H. Marin-Dett, Mateus P. Grejo, Valéria Valente, Mariana P. Palaçon, Andreia Bufalino, Brian M. Peters, Paula A. Barbugli

**Affiliations:** 1 School of Pharmaceutical Sciences, São Paulo State University (Unesp), Araraquara, SP Brazil; 2 School of Dentistry, São Paulo State University (Unesp), Araraquara, SP Brazil; 3 Department of Clinical Pharmacy and Translational Science, College of Pharmacy, University of Tennessee Health Science Center, Memphis, TN 38163 USA

**Keywords:** *Candida albicans*, *Staphylococcus aureus*, Biofilm, Proinflammatory, *TP53*, *BCL2*

## Abstract

Microbes frequently exist as biofilm-embedded multi-species communities where their interactions may establish or exacerbate chronic infection. Recently, fungi and bacteria have been associated with various human tumor microenvironments, suggesting that dynamic cross-kingdom interactions may directly or indirectly contribute to tumor-associated processes. Here, we aimed to investigate whether cell-free supernatants from mono- and dual-species biofilms of the commonly associated fungus *Candida albicans* and bacterium *Staphylococcus aureus* could alter human monocyte responses that promote a tumor-related genetic signature in dysplastic oral epithelial (DOK) cells. Treatment of THP-1 monocytes with *S. aureus* cell-free supernatant increased the production of proinflammatory cytokines (IL-8, IL-1β, and TNF) and CD86 expression. However, exposure to cell-free supernatants from dual-species biofilm suppressed these responses. To determine the fungal virulence factors responsible, *C. albicans* mutants deleted for genes involved in adhesion (*als3*Δ/Δ), hyphal growth (*efg1*Δ/Δ *cph1*Δ/Δ), or candidalysin production (*ece1*Δ/Δ) were assessed during co-culture. While candidalysin was dispensable, loss of hyphal growth or the adhesin Als3p phenocopied effects of *S. aureus* mono-culture treatment. Conditioned medium from THP-1 cells initially challenged with mono- or dual-biofilm cell-free supernatants was applied to DOK cells to assess *TP53* and *BCL2* gene expression. Conditioned medium from *S. aureus* treated THP-1 cells led to decreased epithelial *TP53*, but increased *BCL2* expression, which was reversed by the presence of wild-type *C. albicans*. These phenotypes were similarly dependent on *C. albicans* hyphal growth during dual-biofilm co-culture. Collectively, our results reveal that fungal–bacterial interactions may shape the monocyte–epithelial axis by orchestrating immune responses that enhance tumor-associated gene expression in dysplastic oral epithelial cells.

**Supplementary Information:**

The online version contains supplementary material available at 10.1007/s00430-026-00880-4.

## Introduction

Biofilms formed by bacteria, fungi, and mixed microbial communities are key drivers of chronic and persistent infections, accounting for over 80% of these cases [1, 2]. The chronicity of these surface-attached organisms is underscored by their frequent recalcitrance to conventional therapies due to both genetically encoded drug resistance mechanisms and increased phenotypic tolerance imparted by their complex spatial architecture and thick exopolymeric matrices. Aside from causing overt infectious and inflammatory episodes, recent work has implicated the presence of such biofilm-forming microorganisms, and especially fungi, in tumor microenvironments across diverse human tissues. Moreover, unique tumor-associated bacterial consortia clustered to specific fungal “mycotypes”, suggesting that potentially dynamic cross-kingdom interactions may contribute to dysregulated cell growth and immunologic escape of developing tumors [3].

Chronic inflammatory processes are considered an important marker of cancer development and progression. Bacterial and fungal likely play a role in this regard, considering their capacity to trigger inflammatory pathways, promote genotoxicity, and cause tissue damage [4]. Carcinogenesis can be initiated by microbes broadly via three major steps. First, they must attach to establish colonization of the intratumor environment, primarily by adhesin-ligand interactions. They may then directly interact with immune cells in the tumor microenvironment (TME) to potentially evade detection. Microorganisms can also secrete metabolites and toxins directly within the TME or they may be delivered in an endocrine fashion to non-adjacent sites, traveling through the circulatory or lymphatic systems. Each of these mechanisms can activate pattern recognition receptors (PRRs), elicit inflammatory signaling, and damage tissue to modulate tumor cell physiology [5, 6].

A clear role for extracellular microbes in driving cancer progression is perhaps best understood in the gastrointestinal tract. *Helicobacter pylori* is a known risk factor for gastric cancer [7]. *H. pylori* colonization can drive persistent inflammation of the stomach lining, increased DNA damage, and intestinal metaplasia [7]. In the oral cavity, *Porphyromonas gingivalis* and *Fusobacterium nucleatum* can drive synergistic chronic inflammation to promote carcinogenesis [8, 9]. Aside from secondary metabolites (*e.g.*, gliotoxin, aflatoxin) produced by some molds, fungal-derived pro-tumoral factors have not been extensively described [10]. Given that over 600 bacterial and 100 fungal species exist in the human oral cavity, important new microbe-microbe interactions that impact tumorigenesis remain undiscovered.

Our laboratories have long investigated the interaction between *Candida albicans* and *Staphylococcus aureus*. These microorganisms interact through multiple mechanisms, including physical attachment, metabolic signaling that rewires *S. aureus* toxicity, and modulation of host immune response, facilitating immune-evasion mechanisms [11, 12]. In immunocompromised individuals, oropharyngeal candidiasis has been shown to facilitate the invasion and dissemination of *S. aureus* [13]. Also, *C. albicans* and *S. aureus* are common members of the normal human oral microbiome [14]. The oral mucosal and supragingival surfaces, dental prostheses, and periodontal pockets serve as reservoirs for both species, and their co-isolation is frequently reported in recalcitrant infections [15].

Within this investigative framework, *S. aureus* is known to secrete a range of damaging and inflammatory exotoxins that may disrupt cellular homeostasis [16]. Its presence has been associated with increased production of inflammatory cytokines, including IL-6, IL-1β, and IL-8 [17], and emerging evidence has suggested a potential association between *S. aureus* and oral cancer [18]. Similarly, *C. albicans* produces candidalysin, a pore-forming toxin encoded by the *ECE1* gene, which can drive inflammation, epithelial signaling, and tissue remodeling [19, 20]. The presence of *C. albicans* in oral lesions has also been reported, with its detection correlating with varying degrees of oral epithelial dysplasia [21, 22]. In oral cancer patients, co-occurrence of these microbes has been reported at six months following treatment [23]. Whether such polymicrobial interactions contribute to or promote inflammatory signaling pathways relevant to oral carcinogenesis remains largely unexplored [24]. However, we previously demonstrated that cell-free supernatants from early biofilms (but not those derived from mature biofilms) of *C. albicans* and *S. aureus* modulate the expression of oncogenes (e.g., *PIK3CA*, *HRAS*, *mTOR*, *BRAF* and *BCL2*) in neoplastic oral epithelial cells in vitro [25]. Whether these organisms can also drive epithelial oncogene expression indirectly through manipulation of immune cells commonly found in the oral mucosa has not been explored.

Here, we employed a two-stage in vitro experimental model to answer this question. We investigated whether cell-free supernatant from mono- or dual-biofilms from *C. albicans* and *S. aureus* could influence THP-1 cell responses, and whether this THP-1 conditioned media would, in turn, impact tumor-associated gene expression in DOK cells. While *S. aureus* biofilm cell-free supernatant promoted immune activation markers in THP-1 cells, including CD86 expression and pro-inflammatory cytokine release, the presence of *C. albicans* in dual biofilms suppressed these responses. Use of several *C. albicans* deletion mutants revealed that hyphal formation and Als3p were required, though candidalysin was dispensable, for the suppressive effect. Treatment of DOK cells with THP-1 conditioned media elicited by *S. aureus* biofilm cell-free supernatant resulted in decreased *TP53* and increased *BCL2* gene expression. However, this pattern was abrogated during dual-biofilms of *S. aureus* with wild-type *C. albicans* and partially restored when *S. aureus* was co-cultured with *als3*Δ/Δ and hyphal-deficient mutants. Notably*, S. aureus*–induced gene expression changes partially overlapped with those observed in Detroit 562 carcinoma cells. Collectively, our results suggest that fungal–bacterial interactions modulate the monocyte–epithelial axis, shaping inflammatory responses that may influence gene expression patterns associated with oral dysplasia.

## Materials and methods

### Strains and microorganism growth

*Staphylococcus aureus* strain ATCC 25923 was purchased from Plastilabor (Plastilabor; Rio de Janeiro, RJ, Brazil) and maintained in 20% glycerol stocks at -80 °C. For this study, *S. aureus* was reactivated in Brain Heart Infusion (BHI; Neogen; Lansing, Michigan, USA) agar plates by spreading and incubated at 37 °C for 24 h. For the liquid culture of *S. aureus*, the Trypticase Soy Broth (TSB; Neogen; Lansing, Michigan, USA) media was used. *Candida albicans* WT SC5314, *ece1*Δ/Δ, *als3*Δ/Δ, and *efg1*Δ/Δ *cph1*Δ/Δ were used as described [26]. The *C. albicans* strains were maintained in 20% glycerol stocks at -80 °C. They were routinely grown on Sabouraud Dextrose Agar (SDA; Neogen; Lansing, Michigan, USA) plates at 30 °C for 48 h or liquid Yeast Peptone Dextrose 20 g/L (YPD, Yeast Extract; Neogen; Lansing, Michigan, USA, Peptone; HiMedia; Mumbai, Maharashtra, India, Dextrose; Synth, Diadema, Sao Paulo, Brazil).

### *Candida albicans* and *Staphylococcus aureus* mono- and dual-species biofilm formation

For the mono-species *C. albicans* biofilm, one colony was inoculated in 5.0 mL of fresh YPD and incubated for 16 h at 30 °C. For the inoculum formation, after 16 h, the culture was diluted 1:10 in fresh YPD media and incubated for another 6 h. After the incubation, the optical density (OD) was measured to obtain the mid-log phase (OD_540 nm_: 0.580). The inoculums were standardized to 5 × 10^6^ CFU/mL. The cells were centrifuged at 3,200 x*g*, at 4 °C for 10 min. The cell pellet was washed in Phosphate-Buffered Saline (PBS; 1.37 M NaCl, 27 mM KCl, 100 mM Na_2_HPO_4_, 18 mM KH_2_PO_4_) and resuspended in 10 mL of Roswell Park Memorial Institute 1640 media (RPMI, Sigma-Aldrich; Saint Louis, Missouri, USA) supplemented with HEPES (25 mM; pH: 7.0). After that, 2.0 mL of the inoculum was added into a 75 cm^2^ cell culture flask containing 18 mL of fresh RPMI-1640 media. The culture was incubated for 90 min (min) at 37 °C and 50 rpm for the adhesion phase. After that, it was washed once with 1X PBS and 30 mL of fresh RPMI 1640 media was added and incubated statically for 36 h at 37 °C. For *S. aureus* mono-species biofilm, one colony was inoculated in 10 mL of TSB media and incubated for 16 h at 37 °C. For the inoculum formation, after 16 h, the culture was diluted 1:100 with fresh TSB media and incubated for another 6 h. After the incubation, the OD was measured to obtain the mid-log phase (OD_600 nm_: 0.650). The inoculum was standardized to 5 × 10^7^ CFU/mL. The culture was centrifuged at 3,200 × *g* for 10 min at 4 °C. The cellular pellet was washed in 1X PBS and resuspended in 10 mL of RPMI 1640. After that, 2.0 mL of the inoculum was added into a 75 cm^2^ cell culture flask containing 18 mL of fresh RPMI-1640 media. The culture was incubated for 90 min at 37 °C and 50 rpm for the adhesion phase. After that, it was washed once with PBS 1X and 30 mL of fresh RPMI 1640 media was added and incubated statically for 36 h at 37 °C. For the dual-species biofilms, 2 mL of each inoculum (*S. aureus* and the designated *C. albicans* strain) were added to a 75 cm^2^ flask containing 18 mL of RPMI 1640 for the adhesin phase (90 min). The following steps were performed as described above.

### Extraction of cell-free supernatant present in mono- and dual-species biofilms of *Candida albicans* and *Staphylococcus aureus*

After 36 h, the mono- and dual-species biofilms were collected along with the culture media. Enumeration of colony-forming units (CFU) was performed by a serial dilution of 1:10 by the microdrop plate method. The CFU assay was performed in duplicate on three separate occasions (n = 3). For *C. albicans* growth, the samples were plated in SDA-chloramphenicol (50 mg/L) for 24 h at 30 °C. For *S. aureus*, the samples were plated in BHI-amphotericin B (25 mg/L) for 24 h at 37 °C. The biofilms were filtered using a 0.22 µm SFCA Low-Protein Binding filter (Corning; Oneonta, NY, United States). The total protein amount of the cell-free supernatant was quantified and normalized to 5.53 ± 0.45 µg/mL for mono-cultures of *C. albicans* strains, 12.28 ± 0.18 µg/mL for mono-cultures of *S. aureus,* and 12.30 ± 3.59 µg/mL for dual-species co-cultures, based on previously published research from our group [27]. The protein quantification was performed by Bradford assay (Bradford reagent, Sigma-Aldrich; Saint Louis, Missouri, USA), following the manufacturer's recommendations, in duplicate, on three biological replicates (n = 3).

### Confocal laser scanning microscopy (CLSM)

First, the mono- and dual-species biofilms were formed in SPL Confocal Dishes 35 × 10 mm (SPL Life Science, Gyeonggi-do, South Korea) for 36 h, as described above. Cultures were then stained using the LIVE/DEAD® BacLight Bacterial Viability Kit (Molecular Probes; Eugene, Oregon, United States). Briefly, Syto 9 and Propidium Iodide (PI) were diluted in 1X PBS (1:1000). Then, 3 mL of the diluted solution was added to each Confocal Dish and incubated for 30 min, washed with 1X PBS, and imaged. CLSM was performed using a Carl Zeiss LSM 800 with AiryScan (Carl Zeiss, Jena, Germany). The CLSM setup used a 488 nm laser source (4.5%) with a 530 nm emission filter for Syto 9 detection and a 610 nm emission filter for PI detection, in the X, Y, and Z axes using a Plan-Apochromat 63x/1.40 Oil DIC M27 objective. The image analysis and thickness quantification were performed using the Zen Blue 2.3 Carl Zeiss Software. The assay was performed on three biological replicates (n = 3).

### Blood agar lysis assay

The assay was performed as already described by Paul et al. [11]. The total protein amount of the mono- and dual-species co-cultures was obtained from the fresh cell-free supernatant derived from 36 h biofilms or planktonic growth, using the acetone precipitation method. Briefly, 1.2 mL of pre-chilled − 20 °C acetone was added per 300 µL of each cell-free supernatant sample. The solution was incubated overnight at − 20 °C. Samples were then centrifuged at 13,000 rpm for 10 min at 4 °C. The liquid phase was removed, the pellets dried at room temperature, and resuspended in 80 µL of RPMI 1640 media. Aliquots (30 µL) of protein extracts were applied to wells formed in TSA 5% blood agar plates and incubated for 2 days at 37 °C. The plates were photographed, and the diameter was measured using ImageJ software. The assay was performed in duplicate, on three biological replicates (n = 3).

### Cell culture conditions

The cell lines THP-1 (peripheral blood monocyte, from Acute Monocytic Leukemia), SCC-25 (epithelial Squamous Cell Carcinoma from the tongue), and Detroit 562 (epithelial Pharyngeal Carcinoma, derived from metastatic pleural effusion) were purchased from Rio de Janeiro Cell Bank (BCRJ, Rio de Janeiro, RJ, Brazil). The DOK cell line (a non-tumorigenic, mild to moderate dysplasia from the oral cavity) was purchased from Sigma-Aldrich® (Sigma-Aldrich®, St Louis, MO, USA). All the cells were cultured in 75-cm^2^ flasks at 37 °C and 5% CO_2_. For THP-1 cells, RPMI 1640 media supplemented with HEPES [25 mM], 10,000 units of penicillin; 10 mg/mL of streptomycin; and, 25 µg/mL of amphotericin B (stock solution 100 X), 10% of Fetal Bovine Serum (FBS), L-glutamine (2 mM), Sodium Pyruvate (1 mM) and, 0.05 mM of β-mercaptoethanol (Sigma-Aldrich®, St Louis, MO, USA). The SCC-25 cells were cultured in a 1:1 mixture of Dulbecco’s Modified Eagle’s Medium (DMEM) and Ham’s F12 Medium, containing 1.2 g/L of sodium bicarbonate (LonzaTM BioWhittakerTM, BS, Switzerland), 2.5 mM of L-glutamine, and 15 mM of HEPES, supplemented with 400 ng/ml of hydrocortisone (Sigma-Aldrich®, St Louis, MO, USA) and 10% FBS. The Detroit 562 cells were cultured in DMEM with 1.0 g/L of glucose, 1% non-essential amino acids (LonzaTM BioWhittakerTM, BS, Switzerland), and 10% FBS (Sigma-Aldrich®, St Louis, MO, USA). DOK cells were cultured in DMEM with 4.5 g/L of glucose, 2 mM of L-glutamine, 1 mM of sodium pyruvate, 1,500 mg/L of sodium bicarbonate, and 10% of FBS (Sigma-Aldrich®, St Louis, MO, USA) (Table 1).

**Table 1:** *Candida albicans* mutant strains

Strain	Parental	Genotype	References
*ece1*Δ/Δ	SC5314	*ece1*Δ*:*FRT + *ece1*Δ*:*FRT +	[26]
*als3*Δ/Δ	SC5314	*als3*Δ:FRT + Δ*als3*Δ:FRT +	[26]
*efg1*Δ/Δ *cph1*Δ/Δ	*cph1*Δ/Δ	*cph1*Δ*:*FRT + *cph1*Δ*:*FRT + *efg1*Δ*:*FRT + *efg1*Δ*:*FRT	[26]

### THP-1 stimulation

**Fig. 1:**
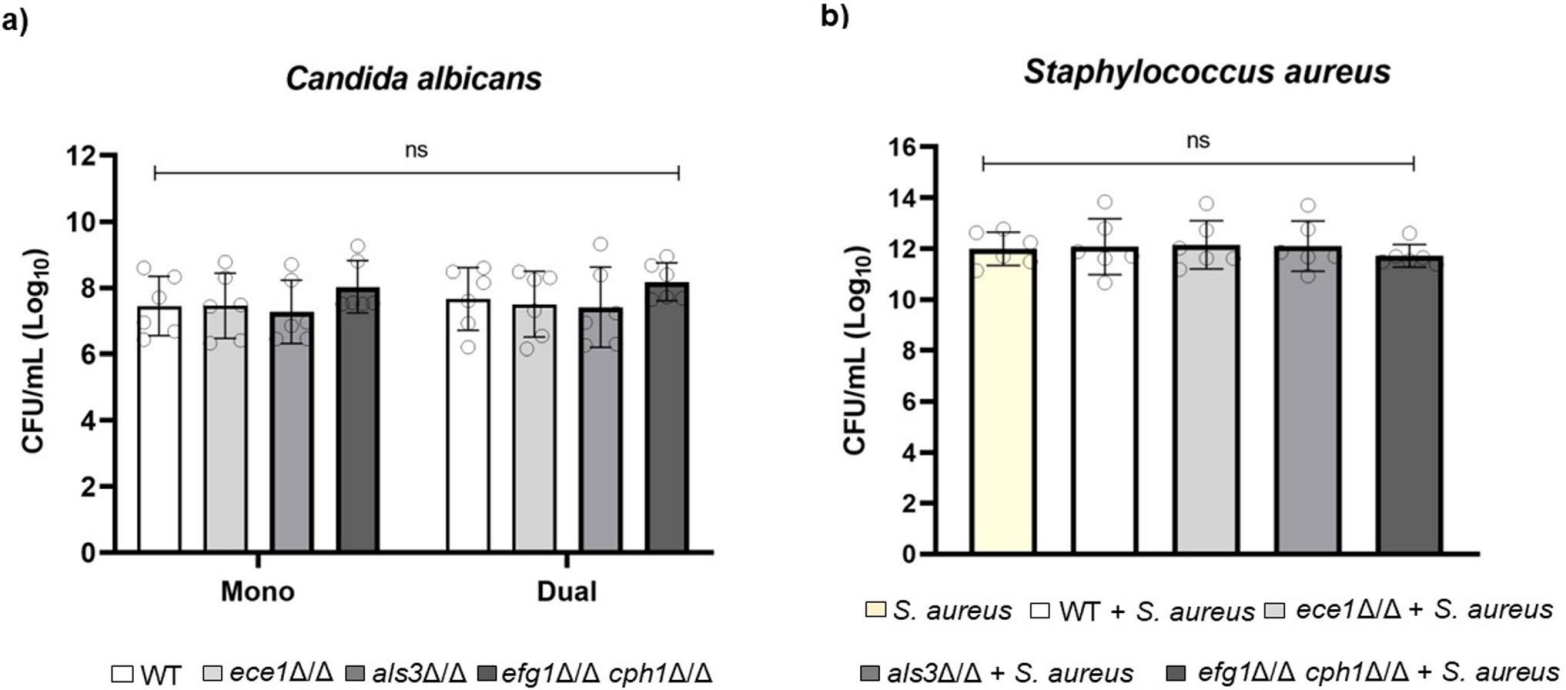
Colony Formation Units per mL (CFU/mL) of 36 h culture growth. **a** Fungal burdens of WT, *ece1*Δ/Δ, *als3*Δ/Δ, and *efg1*Δ/Δ *cph1*Δ/Δ in mono- and dual-species biofilms. Two-way ANOVA followed by Tukey’s post hoc test, ns: not statistically significant. **b** Bacterial burdens of *S. aureus* mono-species and dual-species biofilms, WT + *S. aureus*, *ece1*Δ/Δ + *S. aureus*, *als3*Δ/Δ + *S. aureus,* and *efg1*Δ/Δ *cph1*Δ/Δ + *S. aureus*. One-way ANOVA followed by Tukey’s post hoc test; ns: not statistically significant

First, 5 × 10^6^ THP-1 cells were seeded in a 75 cm^2^ flask containing 24 mL of complete RPMI 1640 media containing FBS 5%, for 16 h at 37 °C and 5% CO_2_. Following, 8.0 mL of biofilm cell-free supernatant was added to each flask (1:4), according to Dias et al. [22]. The cells were incubated for another 24 h at 37 °C and 5% CO_2_. Finally, the conditioned media (CM) were collected and stored at − 20 °C. The remaining THP-1 cells were pelleted by centrifugation, resuspended in 2 mL of RPMI, and distributed into 96-well plates for a 24 h cytotoxicity assay, using the alamarBlue™ reagent, according to the manufacturer's recommendations (Molecular Probes, Eugene, OR, USA). The THP-1 cell cytotoxicity was performed at least in five wells on three biological replicates (n = 3). The total amount of proteins present in CM was measured by Bradford assay, in duplicate, on three biological replicates (n = 3) (Fig. 1. S.M).

### Cell surface receptor labeling assay for CD86 and CD163

First, 3 × 10^5^ THP-1 cells were seeded in a Confocal 35-mm Clear Coverglass-Bottom Petri-Dish (SPL Life Sciences, Pocheon, South Korea) containing 3 mL of complete RPMI 1640 media with FBS 5%, for 16 h at 37 °C and 5% CO_2_. Next, THP-1 cells were stimulated with cell-free supernatant from mono-species *C. albicans*, *S. aureus,* or dual-species biofilms for 24 h. Then, the stimuli were removed, and the cells were independently stained with anti-CD86 Brilliant Violet (BV) 610 (1: 400) (BD-Bioscience; Franklin Lakes, New Jersey, USA) or anti-163 Phycoerythrin–Cyanine Fluor (PE-CF) 594 (1: 200) (BD-Bioscience; Franklin Lakes, New Jersey, USA) antibodies. Cells were incubated for 30 min at 4 °C, carefully washed in 1X PBS, and visualized by CLSM 800 Carl Zeiss. For a positive control, THP-1 cells were treated for 48 h with 100 ng/mL of phorbol12-myristate 13-acetate (PMA; Sigma-Aldrich, St. Louis, MO, United States) followed by a resting phase [28, 29]. The Negative controls included THP-1 cells not stimulated with either cell-free supernatant or PMA and similarly stained for CD86 or CD163 as described above. The negative control was used to establish the baseline fluorescence, which was set to zero. All experimental samples were acquired using identical imaging parameters, and fluorescence intensity was analyzed relative to this baseline. For BV 610 anti-CD86, the CLSM setup was 800 V, using a 405 nm laser source (15%) and a 610 nm detection emission filter. For PE-CF 594 anti-CD163, the CLSM setup was 800 V, using a 561 nm laser source (13%) and a 610 nm detection emission filter. All the cells were imaged using an EC Plan-Neofluor 40x/1.30 Oil DIC M27, scanning 7 × 7 tiles in random areas (a total of 49 fields were acquired per sample). The fluorescence quantification was performed using Zen Blue 2.3 Carl Zeiss software, by Intensity Mean Value. The assay was performed on three biological replicates (n = 3).

### Cytometric bead array assay

THP-1 cells stimulated with cell-free supernatant were evaluated for inflammatory cytokine production with the Human Inflammatory Cytokine Cytometric Bead Array (CBA) Kit (BD Bioscience, San Diego, CA, USA) according to the manufacturer’s protocol. Capture beads were specific for the inflammatory cytokines IL-12p70, TNF, IL-10, IL-6, IL-1β, and IL-8, and incubated with 50 μL of sample. Standard beads for each cytokine were also prepared. Following incubation, 200 µL of Wash Buffer was added to each assay tube to resuspend the bead pellet. Fluorescence measurements were captured using the BD FACS Aria Fusion II (BD Biosciences, San José, CA, USA), recording 5,000 events per sample within the established gate. The data were recorded using BD FACSDiva Software V8.0.1 (BD Biosciences, San José, CA, USA). The data was analyzed by BD FCAP Array V3.0 Software (BD Biosciences, San José, CA, USA). The assay was performed in triplicate, on three biological replicates (n = 3).

### DOK cells stimulation

The dysplastic oral cells (DOK cell line) were stimulated using the THP-1 conditioned media (CM), previously stimulated with cell-free supernatant obtained from mono- and dual-biofilms of *C. albicans* and *S. aureus.* For this, 250,000 cells per well were cultured in a 6-well plate and incubated for 16 h at 37 °C and 5% CO_2_, allowing the cells to attach. The media was removed, cells washed once with 1X PBS, followed by stimulation with 4.0 mL of undiluted CM for 24 h at 37 °C and 5% CO_2_. Two additional controls were performed. DOK cells were stimulated with THP-1 CM not previously incubated with cell-free supernatant (termed THP-1/+) and DOK cells were kept under standard cell culture conditions without receiving any CM (termed THP-1/−). Experiments were performed in triplicate on three biological replicates (n = 3) and RNA extracted for qRT-PCR studies as described below.

### RNA extraction and quantitative RT-PCR

The keratinocyte cell lines, DOK, SCC 25, and Detroit 562 were handled as described by Amaya Arbeláez et al. [25]. First, the total ribonucleic acid (RNA) was isolated using TRIzol® Reagent (Invitrogen™, Thermo Fischer Scientific Inc., MA, USA) according to the manufacturer’s protocol. The concentration and purity of the samples were assessed using NanoDrop™ 2000 (Thermo Fisher Scientific Inc., MA, USA). The mean ratio value of A260/A280 for all RNA samples was 2.01 (± 0.02), reflecting high purity. The RNA was reverse transcribed into cDNA using the High-Capacity cDNA Reverse Transcription kit (Applied Biosystems®, CA, USA). The relative mRNA expression was quantified using real-time PCR analysis in the Gene Amp® 7500 Sequence Detection System (Applied Biosystems®, CA, USA). Amplification products were detected with SYBR Green PCR Master Mix (Applied Biosystems®, CA, USA). HPRT was used as the housekeeping gene [30]. The assay was performed in triplicate on at least two independent assays (n ≥ 6). The relative expression level (Fold Change) was calculated by adopting the 2^−ΔΔCt^ method [31], by comparing to the control group RPMI THP-1/+. Gene expression was also normalized to HPRT by calculating 2^−ΔCt^. Oligonucleotides were designed using the following criteria using OligoExplorer 1.2 software: Tm (melting temperature) of 62–64 °C, GC% content of 55%–60%, 3 'tail GC% content of 40%, and a maximum size of the amplified sequence of 90–150 nucleotides. The oligonucleotide sequences (Table 2) were synthesized by Integrated DNA Technologies, Inc. (IDT-Coralville, IA, USA). The assay was performed in triplicate on three biological replicates (n = 3).

**Table 2:** Oligonucleotide sequences

Gene	Forward	Reverse	References
*HPRT*	TGAGGATTTGGAAAGGGTGT	GAGCACACAGAGGGCTACAA	[30]
*TP53*	GCTGAATGAGGCCTTGGAAC	TTATGGCGGGAGGTAGACTG	[25]
*BCL2*	CAACATCGCCCTGTGGATGA	GCCGTACAGTTCCACAAAGG	[25]

*HPRT* (Hypoxanthine Phosphoribosyltransferase 1), *TP53* (Tumor Protein p53), *BCL2* (B-cell lymphoma 2)

### Statistical analysis

All analyses were performed using GraphPad Prism® version 8.0 software (GraphPad Software Inc., La Jolla, CA, USA). Technical triplicates were averaged within each independent experiment. Statistical analyses were performed using the means from three independent experiments (n = 3). Statistical analyses of the CFU, Bradford and alamarBlue™ assay were performed using an analysis of variance test (Two-Way and One-Way ANOVA) followed by Tukey's post hoc test. For the CLSM, CBA and Quantitative RT-PCR assay, the One-way ANOVA was applied, followed by Dunnett's post hoc test. The significance level adopted for statistical tests was set at 5% (*P* < 0.05).

## Results

### *S. aureus* and *C. albicans* are capable of mutual growth independently of key fungal virulence determinants

As shown previously, *S. aureus* and *C. albicans* are capable of forming symbiotic dual-species biofilm co-cultures [32]. An established in vitro model was used to determine whether bacterial or fungal burdens in mono- and dual-species 36 h biofilms differed between those formed with *C. albicans* wild-type (WT) SC5314, *ece1*Δ/Δ (candidalysin deficient)*, als3*Δ/Δ (hypha adhesin deficient)*,* and *efg1*Δ/Δ *cph1*Δ/Δ (hyphal growth defective)—key effectors of *C. albicans* virulence [12, 33]. Fungal loads from *C. albicans* mono-species biofilms were not significantly different across strains or between those of dual-species biofilms (Fig. 1a). Similarly, staphylococcal burdens were alike during mono- and dual-biofilms cultures with WT and *C. albicans* mutants **(**Fig. 1b**)**. These results indicate that neither *C. albicans* nor *S. aureus* reciprocally influences growth capacity during 36 h dual-species biofilm formation. Somewhat surprisingly, loss of the hyphal adhesin Als3p and capacity to form hyphae, as well as candidalysin expression, did not impact fungal or bacterial loads in this model system.

### Loss of Als3p and hyphal growth impacts mono- and dual-species biofilm architecture and microbial interactions

**Fig. 2:**
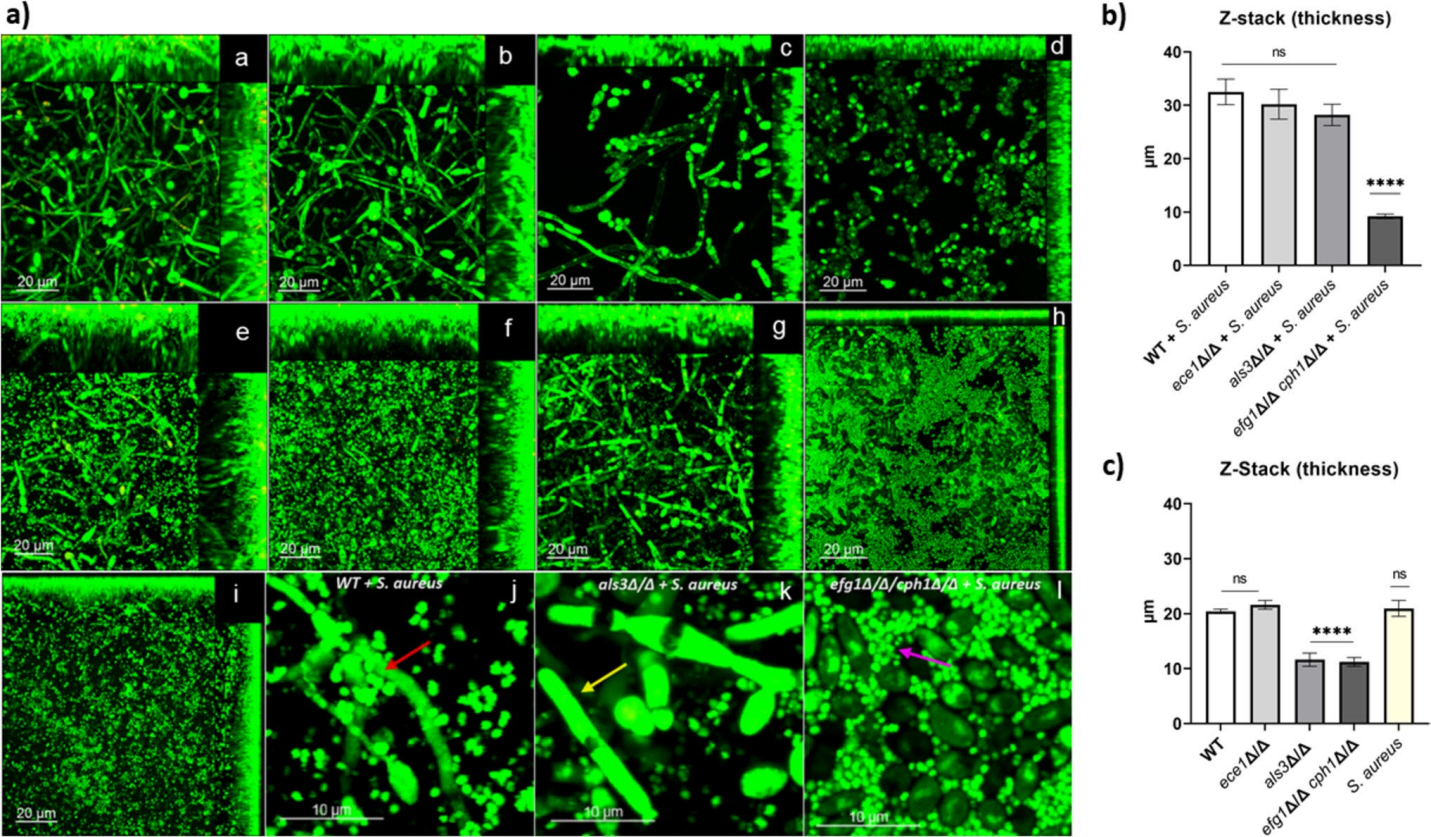
Confocal laser scanning microscopy of 36 h mono- and dual-species biofilms. **a** Morphological features and interactions (a) WT, (b) *ece1*Δ/Δ, (c) *als3*Δ/Δ, (d) *efg1*Δ/Δ *cph1*Δ/Δ, (e) WT + *S. aureus*, (f) *ece1*Δ/Δ + *S. aureus*, (g) *als3*Δ/Δ + *S. aureus*, (h) *efg1*Δ/Δ *cph1*Δ/Δ + *S. aureus*, (i) *S. aureus*, (j) *S. aureus* attached to WT hyphae in red arrows, (k) No *S. aureus* attachment to *als3*Δ/Δ hyphae in yellow arrow and (l) *S. aureus* surrounding *efg1*Δ/Δ *cph1*Δ/Δ yeast cells (pink arrow). **b** Quantification of thickness, in Z-stack axis, from *C. albicans* and *S. aureus* 36 h dual- specie biofilms. One-way ANOVA followed by Dunnett's post hoc test (reference WT + *S. aureus* group), ****P < 0.0001, ns: not statistically significant. **c** Quantification of thickness, in Z-stack axis, from *C. albicans* and *S. aureus* 36 h mono-species biofilms. One-way ANOVA followed by Dunnett's post hoc test (reference WT group), ****P < 0.0001, ns: not statistically significant

Beyond growth capacity, mono- and dual-species biofilms were also evaluated for their morphological features using confocal laser scanning microscopy (CLSM) analysis. *C. albicans* WT formed comparatively thick biofilm architecture during both mono- and dual-species biofilm with *S. aureus* (Fig. 2a.a, a.e, b and c). Mono- and dual-species biofilm formed by the *ece1*Δ/Δ mutant did not display major differences in biofilm architecture and thickness (Fig. 2a.b, a.f, and c). Given its role as an important adhesin during biofilm formation, the *als3*Δ/Δ mutant unsurprisingly displayed a less dense and noticeably reduced thickness of mono-species biofilm (Fig. 2a.c), while dual-biofilm did not display significant differences regarding WT and *ece1*Δ/Δ dual-biofilms (Fig. 2a.g, b and c). Due to its inability to form hyphae, the *efg1*Δ/Δ *cph1*Δ/Δ mutant was unable to establish true biofilms in either mono- or dual-species co-cultures (Fig. 2a.d and a.h). However, it did form an adherent multicellular layer. The mono culture of *S. aureus* biofilm (Fig. 2a.i) was equal to those formed by mono cultures of WT and *ece1*Δ/Δ, and thicker than any of the *efg1*Δ/Δ *cph1*Δ/Δ cultures (Fig. 2b and 2c). Similar to prior studies, enlarged images showed that *S. aureus* attached to WT hyphae (Fig. 2a.j, red arrow), failed to adhere to *als3*Δ/Δ hyphae (Fig. 2a.k, yellow arrows), and coexisted amongst yeast cells of the *efg1*Δ/Δ *cph1*Δ/Δ mutant (pink arrow, Fig. 2a.i).

### *C. albicans *differentially alters *S. aureus* hemolytic activity during planktonic and biofilm co-culture

**Fig. 3:**
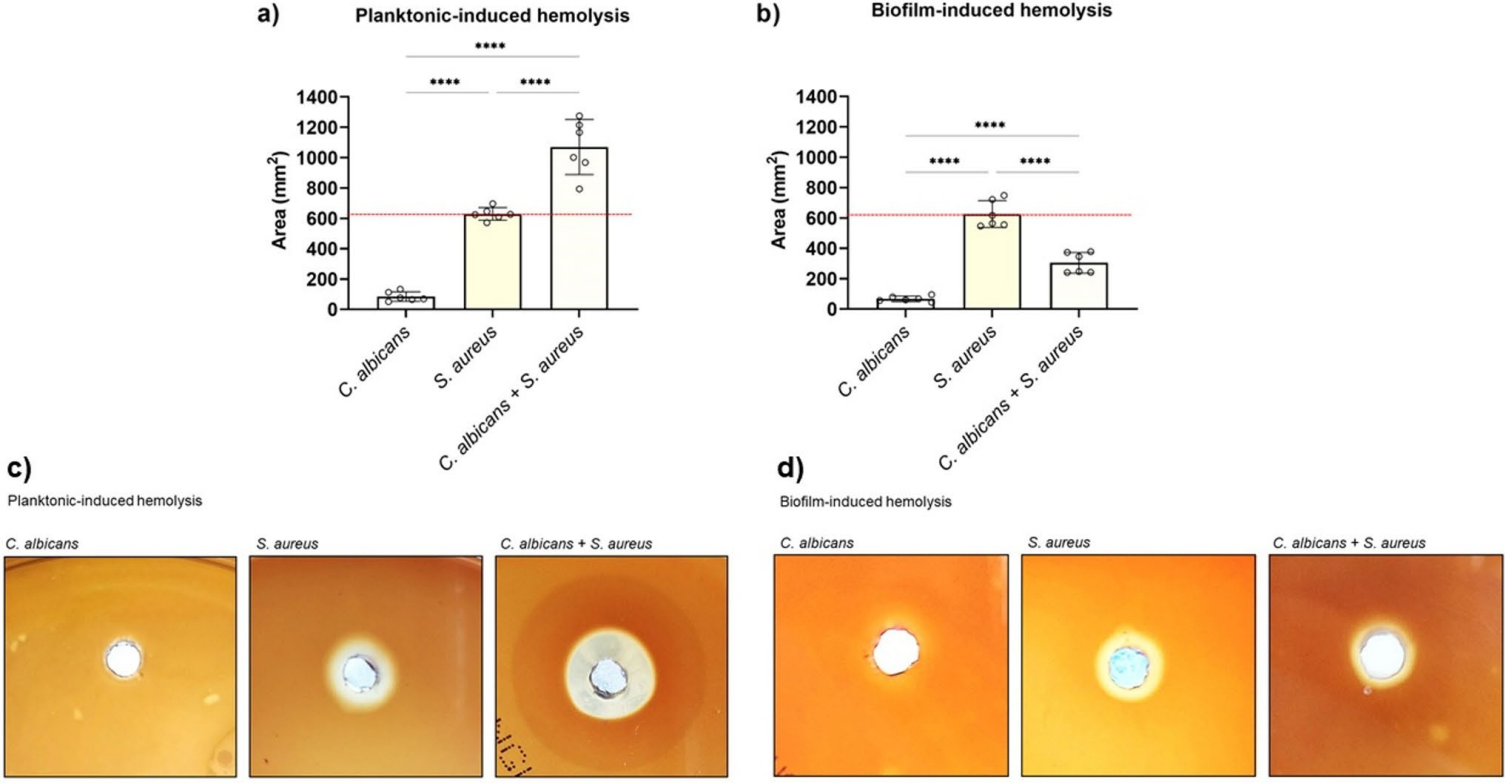
Hemolysis assay performed with planktonic and biofilm protein extracts from mono- and dual-species *C. albicans* and *S. aureus* cultures. **a** Comparison of the area of hemolysis induced by planktonic cultures of mono- and dual-species *C. albicans* and *S. aureus*. ANOVA one-way followed by a Tukey’s post-hoc test, ****P ≤ 0.0001. **b** Comparison of the area of hemolysis induced by biofilm mono- and dual-species of *C. albicans* and *S. aureus*. ANOVA one-way followed by a Tukey’s post-hoc test, ****P ≤ 0.0001. **c** Imaging of hemolytic activity induced by planktonic cultures in TSA 5% blood plates. **d** Imaging of hemolytic activity induced by biofilm in TSA 5% blood plates

*S. aureus* produces a litany of exotoxins that play key and distinct roles in its pathogenesis [34]. Hemolysis on sheep blood agar is primarily driven by the activity of α-hemolysin (Hla) and β-hemolysin (Hlb). Prior work has shown that during planktonic co-culture, the hemolytic capacity of *S. aureus* is enhanced by *C. albicans* via a staphylococcal quorum-sensing-dependent mechanism [35]. While these findings were recapitulated during planktonic growth, as a reference condition, the opposite was unexpectedly found during 36 h biofilm co-culture, where the presence of *C. albicans* repressed *S. aureus-*induced hemolysis (Fig. 3).

### Mono- but not dual-*S. aureus* biofilm cell-free supernatant differentiates naïve THP-1 cells predominantly toward a M1 phenotype

**Fig. 4:**
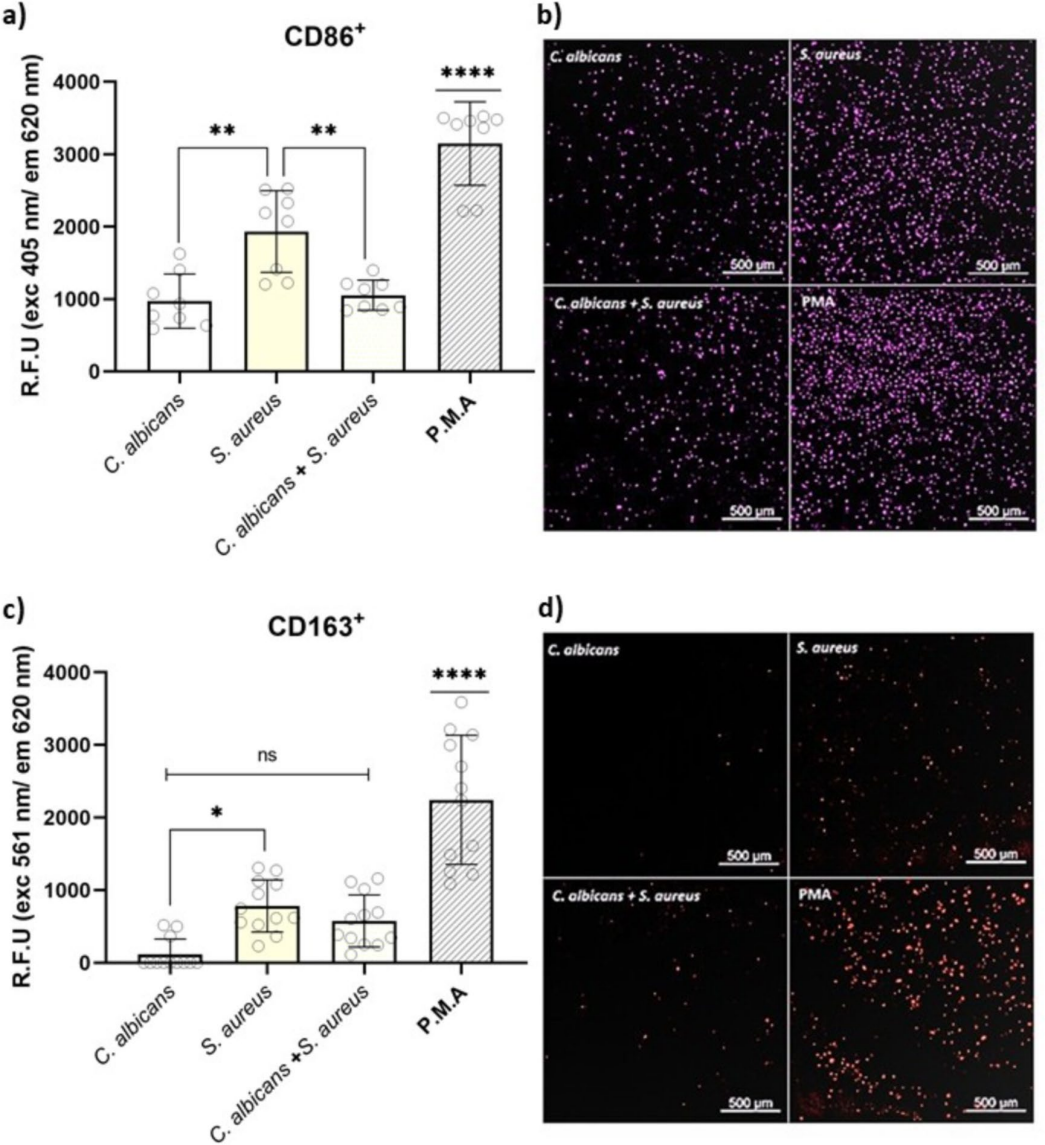
Quantification and imaging by CLSM of CD86+ and CD163+ THP-1 cells. The Relative Fluorescence Units (R.F.U) of (a) CD86+ Brillant Violet 605-conjugated, of THP-1 cells stimulated for 24 h with C. albicans, S. aureus, and C. albicans + S. aureus biofilm cell-free supernatant, and the positive control of THP-1 cells differentiated with PMA. (b) Quantification and imaging by the violet fluorescence of 7x7 CLSM tiles acquisition of random measurements. In (c), CD163+ PE-CF594-conjugated THP-1 cells were stimulated for 24 h with C. albicans, S. aureus, and C. albicans + S. aureus biofilm cell-free supernatant, and the positive control of THP-1 cells differentiated with PMA. (d) Quantification and imaging by the red fluorescence of 7x7 CLSM tiles, acquisition of random measurements. One-way ANOVA followed by Tukey’s post hoc test, ****P < 0.0001, **P < 0.01 and *P < 0.05, ns: not statistically significant

The monocyte co-stimulatory molecule CD86 plays a key role in macrophage activation and is an established marker of M1 polarized macrophages that secrete pro-inflammatory cytokines like IL-1β and TNF. *S. aureus* virulence determinants, including toxins, can drive M1 macrophage activation while persistent infections typically promote an anti-inflammatory M2-like response [36]. Given that, naïve THP-1 monocytes were challenged with *C. albicans* and *S. aureus* cell-free supernatant to investigate the potential of these biofilms to differentiate monocytes toward M1 (CD86^+^) and M2 (CD163^+^) phenotypes. As a positive control for activation, naïve monocytes were treated with phorbol myristate acetate (PMA) for 48 h, followed by a resting phase, to induce CD86^+^ and CD163^+^. *S. aureus* cell-free supernatant induced a significant increase in CD86 surface staining, similar to treatment with PMA (Fig. 4a). Treatment with *C. albicans* cell-free supernatant resulted in comparatively less CD86 staining. Interestingly, the strong induction of CD86 by *S. aureus* cell-free supernatant was repressed during biofilm co-culture with *C. albicans* (Fig. 4a). *S. aureus* cell-free supernatant from both mono- and dual-species biofilms only modestly increased CD163 surface staining (Fig. 4b). *C. albicans* cell-free supernatant had no impact on CD163^+^ differentiation. Collectively, these results showed that cell-free supernatant from *S. aureus* mono-species biofilm predominantly promoted THP-1 cell differentiation toward a pro-inflammatory M1 phenotype, and this effect drastically diminished during co-culture with *C. albicans*, reflective of similarly impaired hemolysis (Fig. 3b and 3d).

### Hyphal growth and *ALS3* expression during dual-species biofilms underpin reduced pro-inflammatory phenotypes elicited by cell-free supernatants

**Fig. 5:**
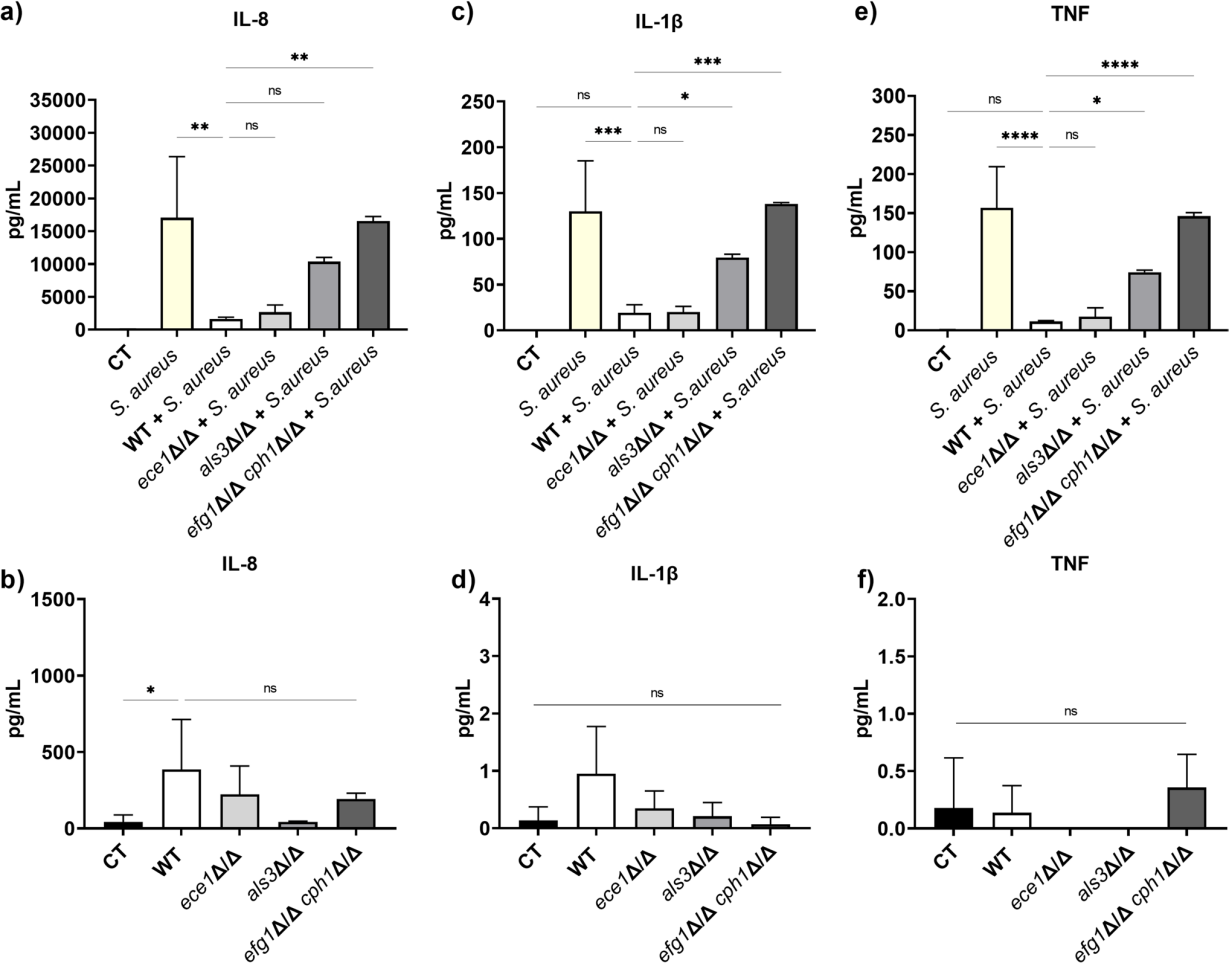
IL-8, IL-1β, and TNF production in naïve THP-1 cells stimulated with mono- and dual-species biofilm-supernatant of C. albicans and S. aureus. (a) IL-8 production from mono- S. aureus and dual-species biofilm cell-free supernatant stimuli. (b) IL-8 production from mono- C. albicans biofilm cell-free supernatant stimuli. (c) IL-1β production from mono- S. aureus and dual-species biofilm cell-free supernatant stimuli. (d) IL-1β production from mono- C. albicans biofilm cell-free supernatant stimuli. (e) TNF production from mono- S. aureus and dual-species biofilm cell-free supernatant stimuli. (f) TNF production from mono- C. albicans biofilm cell-free supernatant stimuli. ****P < 0.0001, ***P < 0.001, **P < 0.01, *P < 0.05 and ns (not statistically significant). For all analyses, One-Way ANOVA was applied, followed by Dunnett's post hoc test, using WT + S. aureus as the reference group (a, c, e) or CT (b, d, f). The results represent cytokines quantification by CBA array using a Flow Cytometer, with 5,000 events per sample.

TLRs, MYD88, and NF-κB signaling pathways are involved in inflammatory responses to *S. aureus* and are associated with the production of the pro-inflammatory cytokines IL-8, IL-1β, and TNF [37]. Similarly, *C. albicans* induces inflammatory responses that terminate in the release of these same cytokines. As cell-free supernatant from *S. aureus* mono-species biofilm drove higher M1 polarization than dual-biofilm cell-free supernatant, levels of IL-8, IL-1β, and TNF were quantified from THP-1 challenged with these cell-free supernatants, to further confirm the M1 phenotype (Fig. 5a, c, e). The levels of IL-6, IL-10, and IL-12p70 were also evaluated, but were not quantifiable. Except for IL-8, cell-free supernatant from mono-species WT biofilm induced negligible levels of IL-1β and TNF (Fig. 5b, d, f). The cell-free supernatant from *S. aureus* mono-species biofilm induced high levels of IL-8, which was inhibited by the presence of WT *C. albicans* in dual-biofilms (Fig. 5a). This inhibition was completely ablated during *S. aureus* co-culture with hyphae-defective mutant *efg1*Δ/Δ *cph1*Δ/Δ (Fig. 5a). The *als3*Δ/Δ showed a tendency to reverse the inhibitory effect induced by the WT strain. However, this effect did not reach statistical significance (*P* = 0.062). The *ece1*Δ/Δ mutant had no appreciable impact on reverting IL-8 production **(**Fig. 5a**)**. Similar findings were revealed for production of the pro-inflammatory cytokines IL-1β (Fig. 5c) and TNF (Fig. 5e). However, the level of these cytokines, induced by cell-free supernatant from dual biofilms of *S. aureus* with *als3*Δ/Δ and *efg1*Δ/Δ *cph1*Δ/Δ mutants were statistically different from the WT co-culture. Importantly, THP-1 cell viability was not impacted by the various cell-free supernatants, and their total protein content was similar, so these phenotypes were not due to potential impacts on cell survival (Online Resource 1). As *S. aureus* was previously shown to physically interact with *C. albicans* hyphae via the adhesin Als3p, our results suggest that such interactions during co-culture biofilms are likely responsible for altering the cell-free supernatant that pushes toward pro-inflammatory differentiation of THP-1 cells.

### THP-1 conditioned medium elicited by cell-free supernatant from *S. aureus* mono-species biofilm downregulates *TP53* expression in DOK cells.

**Fig. 6:**
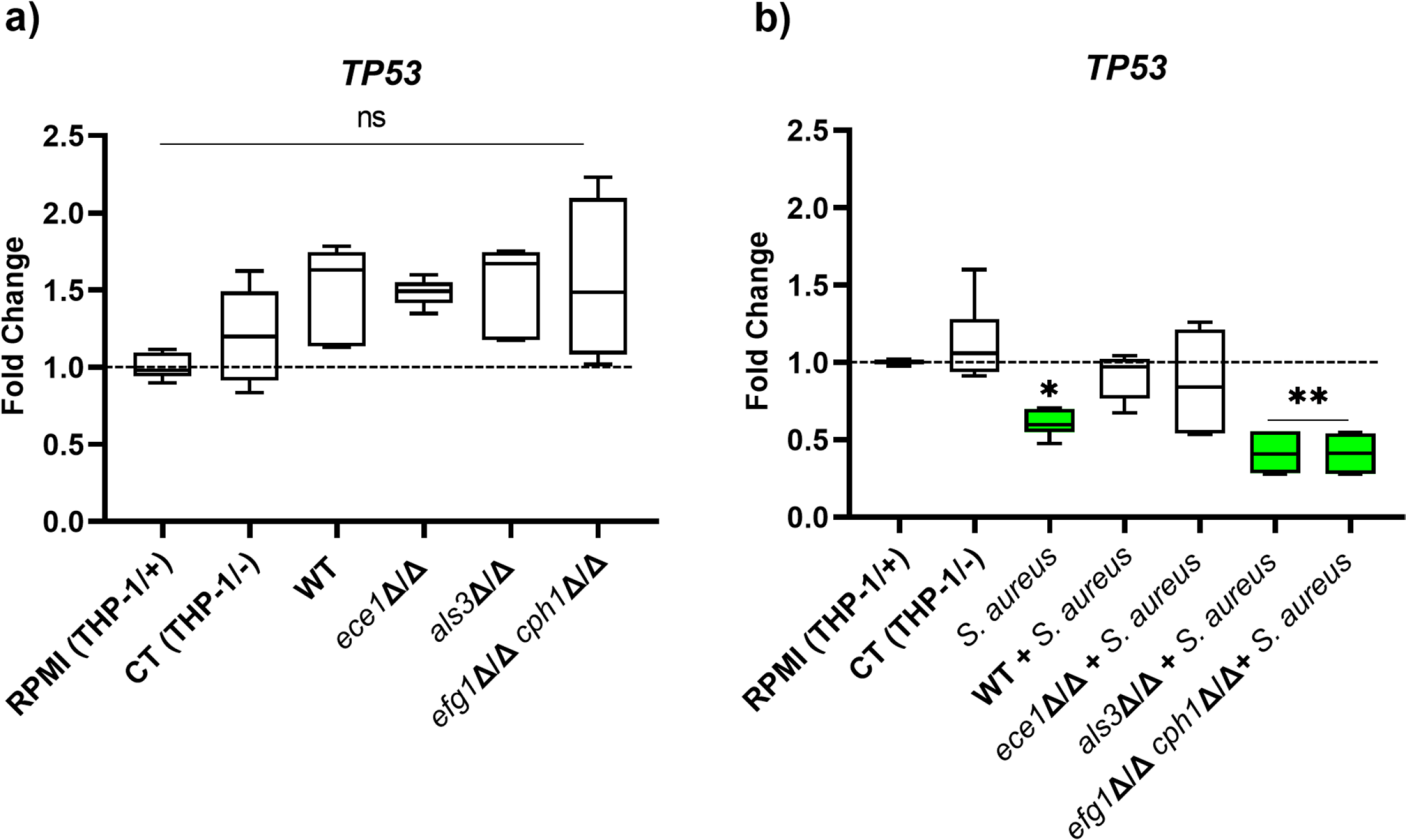
The *TP53* gene expression of DOK cells after 24 h of incubation with CM from naïve THP-1 cells previously stimulated with mono- and dual-species biofilm cell-free supernatant. **a** The DOK fold change (FC); RPMI (THP-1/+): DOK cells stimulated with CM from THP-1 in RPMI, CT (THP-1/-): DOK cells in DMEM, WT: DOK cells stimulated with CM from THP-1 previously stimulated with *C. albicans* WT biofilm cell-free supernatant; *als3Δ/Δ*: DOK cells stimulated with CM from THP-1 previously stimulated with *C. albicans als3*Δ/Δ biofilm cell-free supernatant; *efg1*Δ/Δ *cph1*Δ/Δ: DOK cells stimulated with CM from THP-1 previously stimulated with *C. albicans efg1*Δ/Δ *cph1*Δ/Δ cell-free supernatant. **b** The DOK fold change (FC); RPMI (THP-1/+): DOK cells stimulated with CM from THP-1 in RPMI, CT (THP-1/-): DOK cells in DMEM, *S. aureus*: DOK cells stimulated with CM from THP-1 previously stimulated with *S. aureus* biofilm cell-free supernatant; WT + *S. aureus*: DOK cells stimulated with CM from THP-1 previously stimulated with *C. albicans* WT + *S. aureus* biofilm cell-free supernatant; *als3Δ/Δ* + *S. aureus*: DOK cells stimulated with CM from THP-1 previously stimulated with *C. albicans als3*Δ/Δ + *S. aureus* biofilm cell-free supernatant; *efg1*Δ/Δ *cph1*Δ/Δ + *S. aureus*: DOK cells stimulated with CM from THP-1 previously stimulated with *C. albicans efg1*Δ/Δ *cph1*Δ/Δ + *S. aureus* cell-free supernatant. One-way ANOVA followed by Dunnett's post hoc test, using RPMI as the reference group, *P < 0.05, **P < 0.01, and ns: not statistically significant

The pro-inflammatory cytokines IL-8, IL-1β, and TNF have been associated with cancer-related inflammatory pathways and tumor progression [36], contributing to cell-to-cell communication within the TME. Sustained pro-inflammatory signaling has been linked to cellular changes, including dysregulation of *TP53* expression. As monocytes are key sentinels of the TME, we investigated whether mono- and dual-species biofilm cell-free supernatants modulate THP-1–mediated inflammatory signaling and subsequently affect *TP53* expression in DOK cells. Treatment of DOK cells, with THP-1 conditioned medium (CM), derived from *C. albicans* mono-species cell-free supernatant did not significantly alter *TP53* expression as compared to unconditioned or fungus-free conditioned medium (Fig. 6a). However, THP-1 CM elicited by mono- species *S. aureus* cell-free supernatant were responsible for a 40% reduction in *TP53* gene expression compared to bacteria-free CM (Fig. 6b). Additionally, THP-1 CM elicited by dual-species cell-free supernatants of *S. aureus* with *als3Δ/Δ* and *efg1Δ/Δ cph1Δ/Δ* mutants reduced *TP53* expression by approximately 60% (Fig. 6b). However, CM elicited by cell-free supernatants from dual biofilms of *S. aureus* with WT and *ece1*Δ/Δ strains did not significantly alter expression of *TP53* as compared to the controls (Fig. 6b). These phenotypes were consistent with the inability of *als3*Δ/Δ and *efg1*Δ/Δ *cph1*Δ/Δ mutant cell-free supernatants to suppress cytokine signaling in THP-1 cells (Fig. 4b). The *TP53* is negatively regulated by chronic inflammation and pro-inflammatory cytokines like IL-8. IL-1β and TNF are known activators of angiogenesis, tumor growth, invasion, and metastasis. Within the limits of our in vitro model, our results suggest that *S. aureus* cell-free supernatant may promote inflammatory processes associated with *TP53* downregulation in DOK cells. Additionally, *C. albicans* attenuated this response during co-culture, in an Als3p and hyphae-dependent manner.

### THP-1 conditioned medium elicited by cell-free supernatant from *S. aureus* mono-species biofilm enhances *BCL2* expression in DOK cells

**Fig. 7:**
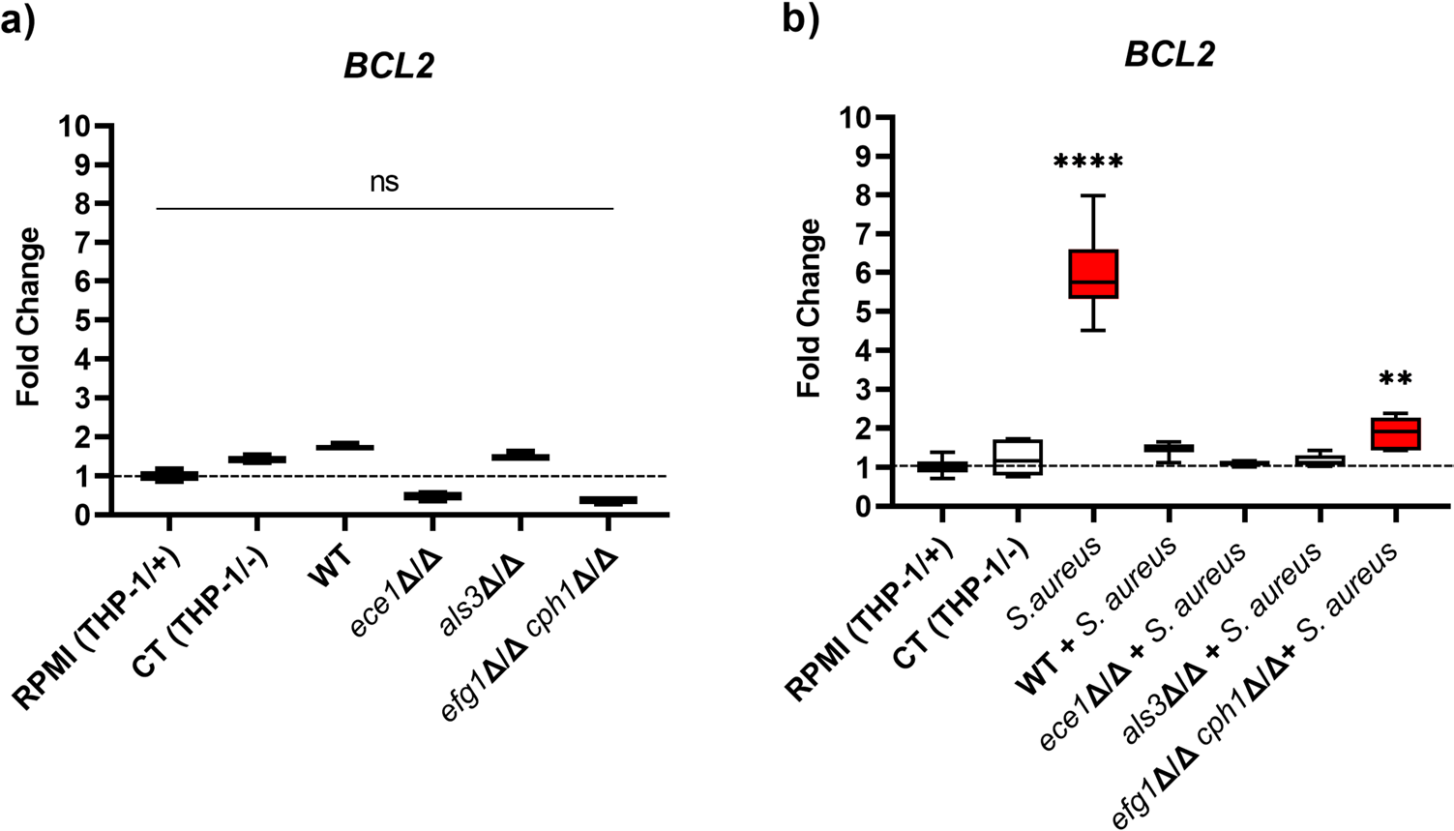
The *BCL2* gene expression of DOK cells after 24 h of incubation with CM from naïve THP-1 cells previously stimulated with mono- and dual-species biofilm cell-free supernatant. **a** The DOK fold change (FC); RPMI (THP-1/ +): DOK cells stimulated with CM from THP-1 in RPMI, CT (THP-1/-): DOK cells in DMEM, WT: DOK cells stimulated with CM from THP-1 previously stimulated with *C. albicans* WT biofilm cell-free supernatant; *als3Δ/Δ*: DOK cells stimulated with CM from THP-1 previously stimulated with *C. albicans als3*Δ/Δ biofilm cell-free supernatant; *efg1*Δ/Δ *cph1*Δ/Δ: DOK cells stimulated with CM from THP-1 previously stimulated with *C. albicans efg1*Δ/Δ *cph1*Δ/Δ cell-free supernatant. **b** The DOK fold change (FC); RPMI (THP-1/+): DOK cells stimulated with CM from THP-1 in RPMI, CT (THP-1/-): DOK cells in DMEM, *S. aureus*: DOK cells stimulated with CM from THP-1 previously stimulated with *S. aureus* biofilm cell-free supernatant; WT + *S. aureus*: DOK cells stimulated with CM from THP-1 previously stimulated with *C. albicans* WT + *S. aureus* biofilm cell-free supernatant; *als3Δ/Δ* + *S. aureus*: DOK cells stimulated with CM from THP-1 previously stimulated with *C. albicans als3*Δ/Δ + *S. aureus* biofilm cell-free supernatant; *efg1*Δ/Δ *cph1*Δ/Δ + *S. aureus*: DOK cells stimulated with CM from THP-1 previously stimulated with *C. albicans efg1*Δ/Δ *cph1*Δ/Δ + *S. aureus* cell-free supernatant. One-way ANOVA followed by Dunnett's post hoc test, using RPMI as the reference group, ****P < 0.0001, **P < 0.01, and ns: not statistically significant

Dysregulated cell proliferation and differentiation are major checkpoints of cancer development. *BCL2* is an anti-apoptotic gene that can broadly facilitate uncontrolled cell proliferation, including in oral dysplastic lesions [38]. Similar to the experiments above, we evaluated whether THP-1 conditioned medium elicited by mono- and dual-species biofilm cell-free supernatants modulates *BCL2* gene expression in DOK cells. The results herein show that the THP-1 CM from *C. albicans* mono-species biofilms did not significantly alter the *BCL2* gene expression (Fig. 7a). In contrast, THP-1 CM elicited by mono-species *S. aureus* biofilm significantly upregulated the *BCL2* gene expression in DOK cells (FC of 5.95, representing a 495% increase in *BCL2* expression). This phenotype was abolished by the presence of WT *C. albicans* (FC = 1.47) but not *als3*Δ/Δ (FC = 1.17), or *ece1*Δ/Δ (FC = 1.10) strains during biofilm co-culture. This effect was marginally, but significantly, reversed during DOK treatment with CM obtained from the cell-free supernatant of *S. aureus* with *efg1*Δ/Δ *cph1*Δ/Δ (FC of 1.87, representing an 87% of increase in *BCL2* gene expression) (Fig. 7b). These results demonstrate that THP-1 CM elicited by *S. aureus* mono-species cell-free supernatant can drive ~ sixfold upregulation of the *BCL2* gene in vitro, and this effect was abolished by the presence of *C. albicans*, in a hyphae-dependent manner.

### *S. aureus* biofilm supernatant indirectly modulates gene expression in DOK cells, with patterns resembling those observed in metastatic pharyngeal carcinoma cells

**Fig. 8:**
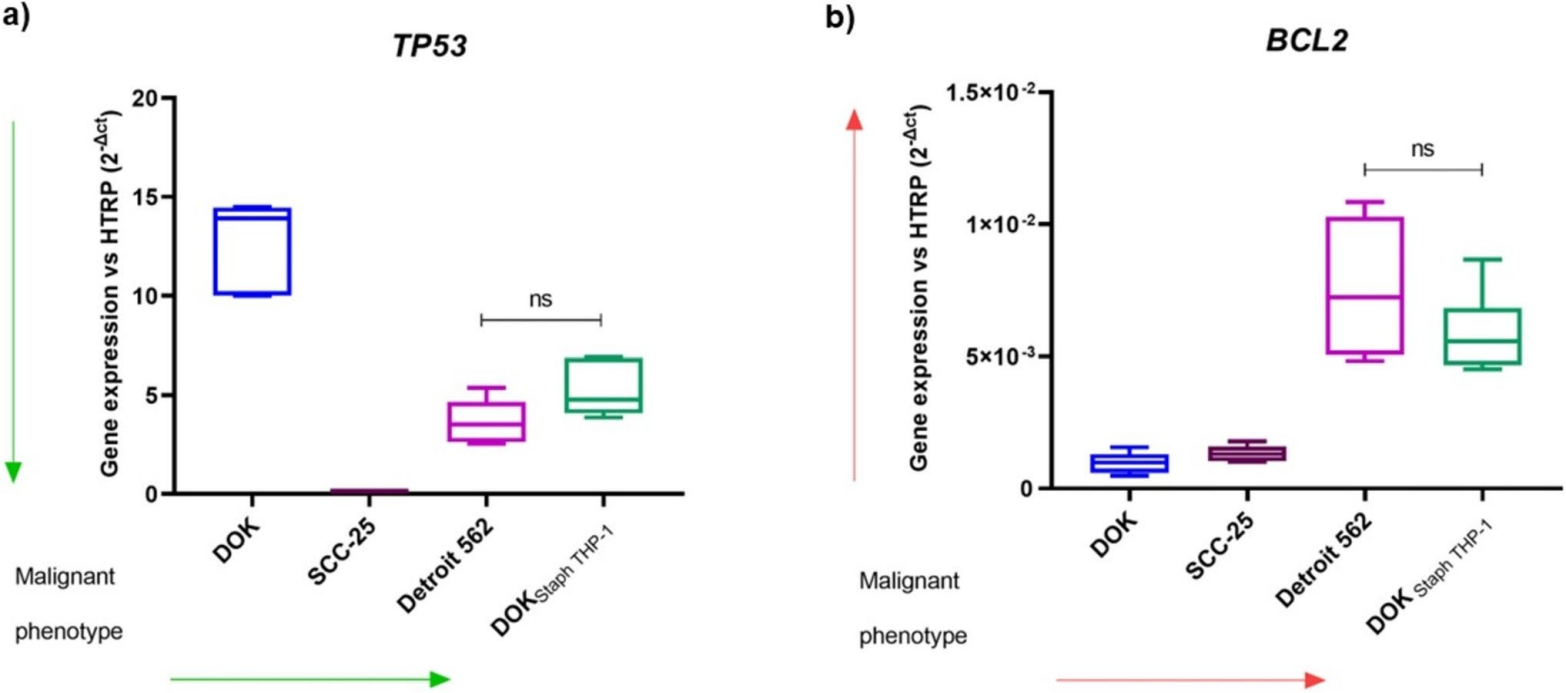
The *TP53* and *BLC2* gene expression of dysplastic oral cells (DOK), squamous cell carcinoma from the tongue (SCC-25), and metastatic pharyngeal carcinoma (Detroit 562). **a** The *TP53* gene expression decreases according to the malignant phenotyping progression. The DOK *TP53* gene expression decreased to the same level as Detroit 562 cells, after 24 h of incubation with CM from naïve THP-1 cells previously stimulated with *S. aureus* biofilm cell-free supernatant (DOK *Staph* THP-1). **b** The *BCL2* gene expression increases according to the malignant phenotyping progression. The DOK *BCL2* gene expression increased to the same level as Detroit 562 cells, after 24 h of incubation with conditioned media from naïve THP-1 cells previously stimulated with *S. aureus* biofilm-supernatant (DOK *Staph* THP-1). One-way ANOVA followed by Tukey’s post hoc test, ns: not statistically significant

The signaling between p53 and Bcl-2 is fundamental in the onset of carcinogenesis, as pro-apoptotic p53 levels are inversely correlated with anti-apoptotic Bcl-2 family members [39]. Thus, the coordinated activity of these factors is a checkpoint to limit tumorigenesis. Dysplastic, but not yet cancerous, DOK cells expectedly displayed relatively high expression of *TP53* (Fig. 8a) and low expression of *BCL2 *(Fig. 8b). Profiling of these markers in squamous carcinoma cell line SCC-25 and more aggressive metastatic pharyngeal carcinoma cell line Detroit 562 revealed significantly lower *TP53* and higher *BCL2* expression, which is consistent with their bona fide cancer phenotypes. Treatment of dysplastic DOK cells with THP-1 CM elicited by *S. aureus* mono-species biofilm cell-free supernatant resulted in a shift in *TP53* and *BCL2* gene expression that resembled the metastatic Detroit 562 profile (Fig. 8a and b). These findings suggest that staphylococcal cell-free supernatant shapes a monocyte-derived inflammatory milieu, associated with modulation of *TP53* and *BCL2* gene expression in dysplastic cells, resembling molecular patterns observed in more aggressive oral carcinoma cells.

## Discussion

Synergistic, antagonistic, mutualistic, opportunistic, and symbiotic relationships between fungal and bacterial species have been previously reported, with many of these governed via quorum-sensing crosstalk and contact-dependent attachment. To this point, the interaction between *C. albicans* and *S. aureus* during co-culture biofilm formation is a prime example of how cross-kingdom communication can shape microbial virulence [32]. The master regulators Efg1p and Cph1p play crucial roles in the yeast-to-hypha morphological transition that is central to *C. albicans* pathogenicity [33, 40]. Aside from driving fungal invasion, hyphae also provide a structural scaffold for the physical attachment of *S. aureus* through the Als3p adhesin [12]. This key attachment step may facilitate more directed release and sensing of interspecies signals.

Another key virulence factor studied herein, candidalysin, the pore-forming *C. albicans* toxin, triggers the host response by promoting cellular damage and immune activation [19]. While hypha formation and Als3p are widely recognized as a critical point of *S. aureus* interaction [12], the potential role of candidalysin in this context has not been extensively explored. Results from this study revealed that during dual biofilms, *S. aureus* displayed no apparent differences in adherence to or growth with the *ece1*Δ/Δ strain compared to wild-type *C. albicans* [41]. A study by Short et al. [42] similarly reported that *S. aureus* robustly attached to hyphae of an *ece1*Δ/Δ mutant and induced few transcriptional changes compared to binding to wild-type *C. albicans*. However, abundant transcriptional changes were noted during co-culture with *als3*Δ/Δ, confirming that Als3p is likely the major adhesin regulating fungal-bacterial attachment and that *C. albicans* is responsive to close bacterial colonization [42].

While impacts of *C. albicans* co-culture on *S. aureus* virulence have been studied during planktonic growth, similar dynamics in co-culture are poorly understood. The hemolytic capacity of *S. aureus*, known to be enhanced in planktonic co-culture with *C. albicans* and in in vivo disseminated infection models [43], is paradoxically inhibited by *C. albicans* in 36 h biofilm co-culture. Consistently, Vila et al. [44] reported the downregulation of key virulence factors (including toxins, gamma-hemolysins, alpha-hemolysin, leucocidin, and staphylokinase) in addition to immunomodulatory and adhesion genes in a catheter-associated biofilm model. This behavior can be due to a downregulation of the anti-holin-like repressors and DNA nuclease (*nuc*), potentially facilitating the upregulation of adhesion-related genes. However, we cannot rule out the biofilm modality itself and the distinct associated regulatory events as critical determinants of this phenotype. In either case, further investigation into these mechanisms is warranted.

It is widely recognized that *C. albicans* and *S. aureus* are potent inducers of host inflammatory pathways. Pattern recognition receptors (PRRs), such as Toll-like receptors (TLRs) 2, TLR4, and Dectin-1, play a pivotal role in recognizing both species [45, 46]. Mainly through TLR2 and NF-κB activation, *S. aureus* can promote the upregulation of co-stimulatory molecules, including CD86, which is crucial for T-cell activation [47]. *S. aureus* typically upregulates CD86^+^ on antigen-presenting cells (APCs), into macrophages or dendritic cells, in response to infection or to exposure of bacterial components such as lipoteichoic acid (LTA), peptidoglycan, hemolysins, protein A, and superantigens [47]. While *C. albicans* can trigger inflammatory responses, primarily via the Dectin-1 pathway [46], Netea et al. [48] described that TLR2 activation by *C. albicans* suppresses antifungal immunity by inducing IL-10 and regulatory T cells (Tregs) [48]. Conversely, Leonhardt et al. [49] describe that several markers of maturation of dendritic cells, including CD86, are significantly reduced in the presence of the *C. albicans* quorum signal farnesol. These opposing immune responses elicited by *C. albicans* and *S. aureus* make it challenging to disentangle their behavior during co-infection, especially within the context of biofilm growth. However, results in this study have begun to clarify these dynamics.

Initially, the maturation of THP-1 naïve monocytes, as determined by CD86^+^ expression, was strongly induced by cell-free supernatant from *S. aureus* mono-species biofilms—comparable to the chemically induced maturation promoted by PMA. Surprisingly, cell-free supernatant from dual-species *C. albicans* and *S. aureus* biofilms reduced THP-1 CD86^+^ cells and also the IL-8, IL-1β, and TNF production, to the same levels as those induced by *C. albicans* mono-species biofilms. This reduction in the monocyte’s polarization and in the antigenic capacity of *S. aureus*, promoted by *C. albicans* presence in dual-species biofilms, was not influenced by the *ECE1* gene, but rather by Als3p, Efg1p, and Cph1p.

In contrast, Pasman et al. [50] reported an upregulation of TNF-related genes in PMA-differentiated macrophage-like THP-1 cells stimulated with the secretome of a dual-species *C. albicans*–*S. aureus* biofilm. A similar response was observed in our previous study [27], in which J774 macrophages stimulated with 36 h dual-species biofilm cell-free supernatant produced TNF levels comparable to those induced by *S. aureus* monocultures and higher than those induced by *C. albicans* monocultures. In contrast, herein, we employed naïve THP-1 monocytes to investigate the direct effects of biofilm-derived cell-free supernatant on monocyte-to-macrophage differentiation, thereby capturing early innate sensing events that may be obscured in PMA-preconditioned macrophage-like models.

In line with these propositions, stimulation of THP-1 cells with cell-free supernatant from mono-species *C. albicans* biofilms resulted in a weak induction of inflammatory responses, producing only levels that could be considered marginally detectable. This suggests that, in this context, the bacterial-fungal coexistence attenuates THP-1 cell polarization and specialization, in a hypha-contact dependent manner and independently of candidalysin. Also, instead of promoting inflammation, the differentiation of monocytes into M2-like cells via CD163^+^ expression has also been associated with *S. aureus*, particularly in specific contexts such as chronic biofilm-associated infections [51]. However, this behavior was not clearly observed in our experimental system, likely due to the short period of THP-1 stimulation, as a limitation of our work.

Fungus- and bacteria-induced inflammatory signaling goes beyond the understanding of infectious disease pathogenesis. Recently, the importance of understanding the role of microorganisms in pre-cancerous and cancer development, progression, and resistance has been well recognized. Although numerous associations have been described clinically, especially in oral and head and neck lesions, it remains unclear whether these correlations are causal for disease progression or are merely a consequence of the tumor presence and the associated immunosuppressive environment [52]. Unraveling the repertoire of secreted molecules released by microbes may provide a robust approach to establishing molecular mechanisms underlying tumor-microbe interactions [53, 54].

The host immune response involves the recognition of microbial components by PPRs, activating various intracellular cascades, leading to cytokine release [55]. In general, cytokines play a crucial role in the immune response of oral dysplasia, promoting cell migration, angiogenesis, invasion, and uncontrolled cell proliferation [56]. In this regard, TNF, IL-1β, and IL-8 have been extensively investigated as key pro-inflammatory stimuli [56]. The induction of IL-8, which can also be directly induced by TLR stimulation, contributes to the epithelial-mesenchymal transition (EMT) important for cellular invasion, migration, and immune evasion during cancer progression [57]. TNF signaling is increased in Oral Potentially Malignant Disorders (OPMDs), in vivo, and in clinical samples [58].

Despite their incidence in the oral cavity, there are relatively few reports linking *C. albicans* and *S. aureus* to the cellular mechanisms underlying OPMDs progression or severity. However, *S. aureus* can activate the COX-2/PGE2 axis in human oral keratinocytes, increasing cellular proliferation. This effect was associated with upregulation of cyclin D1 and downregulation of p16, as well as enrichment of NF-κB and TNF pathways [18]. Notably, COX-2 upregulation was also observed in oral cancer patients [59]. Additionally, a previous report linked *C. albicans* infection to IL-1β secretion during OPMDs [60]. In our in vitro study, cell-free supernatant produced by mono-species *S. aureus* biofilm clearly elicited IL-8, IL-1β, and TNF production from naïve THP-1 cells. This conditioned medium promoted a shift in *TP53* and *BCL2* gene expression in DOK cells, resembling the basal levels of metastatic Detroit 562 cells. Importantly, this genotypic profile is a key signature of certain tumor phenotypes [61–63]. In a manner dependent on hyphal contact, *C. albicans* promoted an immunosuppressive milieu that markedly decreased the *S. aureus*-induced inflammatory response. Thus, it is plausible that *C. albicans* may serve a beneficial role at the oral mucosa by decreasing the release of staphylococcal shed or secreted effectors that indirectly encourage malignant transformation. However, the apparent immunosuppressive impact of *C. albicans* secreted factors may contribute to disease chronicity or anti-cancer immunity through unexplored mechanisms.

In summary, our collective results demonstrate that complex microbe-microbe interactions can orchestrate unpredictable cellular events. These findings support a role for bacterial–fungal interactions in shaping immune-related responses and associated gene expression patterns relevant to tumor biology.

## Supplementary Information

Below is the link to the electronic supplementary material.

Supplementary file1 (PDF 208 KB)

Supplementary file2 (TIF 17992 KB)

## Data Availability

The authors confirm that the data supporting the findings of this study are available within the article and its supplementary materials.
