# Supplementary material for: Candida albicans hyphae modulate Staphylococcus aureus cell-free supernatant during dual biofilm growth to drive molecular signatures of oral dysplasia
Source: Med Microbiol Immunol. 2026 Jun 19;215(1):19. doi: 10.1007/s00430-026-00880-4 (PMC13282323; doi:10.1007/s00430-026-00880-4)
Supplement: Supplementary file 1 — Supplementary file1 (PDF 208 KB) [file 430_2026_880_MOESM1_ESM.pdf]

Online Resource 1 Cellular viability and total amount of proteins present in conditioned medium from THP-1 cells stimulated with biofilm-supernatants.

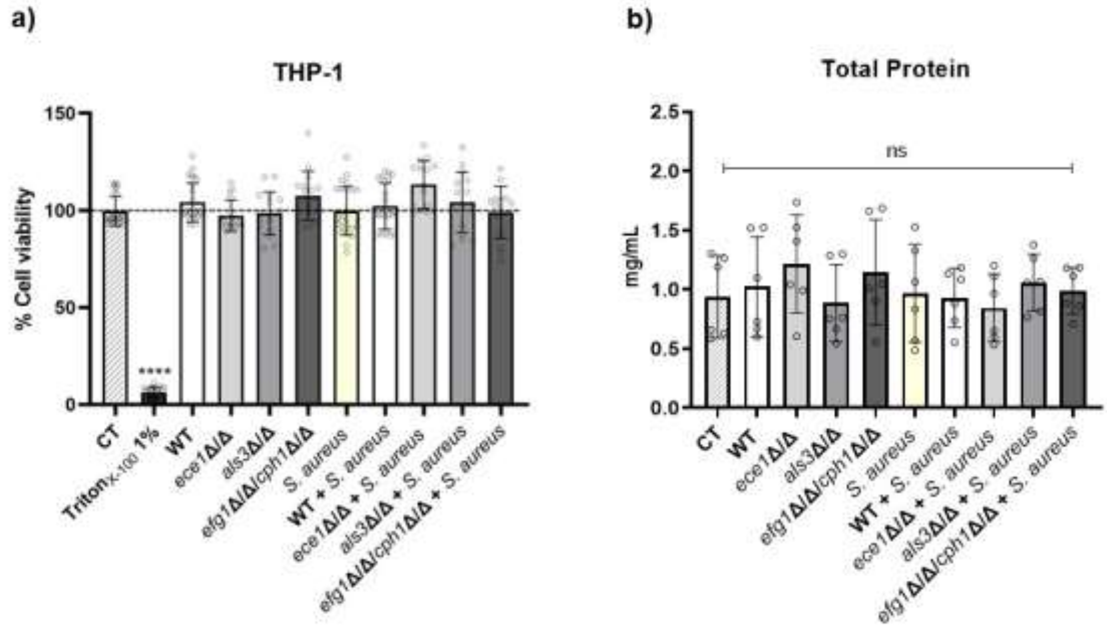

Quantification of the THP-1 cellular viability and total amount of proteins present in conditioned medium (CM) from THP-1 cells. (a) Cell viability by alamarBlue^{TM} of the THP-1 cells stimulated for 24 h with mono- and dual-species *C. albicans* and *S. aureus* biofilm-supernatant. ANOVA One-way followed by a Tukey's post-hoc test, ns: not statistically significant, n ≥ 15. (b) Total protein concentration (mg/mL) by Bradford assay, present in CM of THP-1 cells after 24 h of stimulation with mono- and dual-species *C. albicans* and *S. aureus* biofilm-supernatant. ANOVA One-way followed by a Tukey's post-hoc test, ns: not statistically significant, n = 6.

***Candida albicans* hyphae modulate *Staphylococcus aureus* secreted effectors during co-culture biofilm growth to drive molecular signatures of oral epithelial dysplasia**

Journal: Medical Microbiology and Immunology

Freddy H. Marin-Dett[1], Mateus P. Grejo[1], Valéria Valente[1], Mariana P. Palaçon[2], Andreia Bufalino[2], Brian M. Peters[3], Paula A. Barbugli[1,2*]

[1]São Paulo State University (Unesp), School of Pharmaceutical Sciences, Araraquara, SP, Brazil

[2]São Paulo State University (Unesp), School of Dentistry, Araraquara, SP, Brazil

[3]Department of Clinical Pharmacy and Translational Science, College of Pharmacy, University of Tennessee Health Science Center, Memphis, TN 38163, USA

* **Corresponding author:** Paula Aboud Barbugli, paula.barbugli@unesp.br,
