# Supplementary figures and images for: Candida albicans hyphae modulate Staphylococcus aureus cell-free supernatant during dual biofilm growth to drive molecular signatures of oral dysplasia
Source: Med Microbiol Immunol. 2026 Jun 19;215(1):19. doi: 10.1007/s00430-026-00880-4 (PMC13282323; doi:10.1007/s00430-026-00880-4)

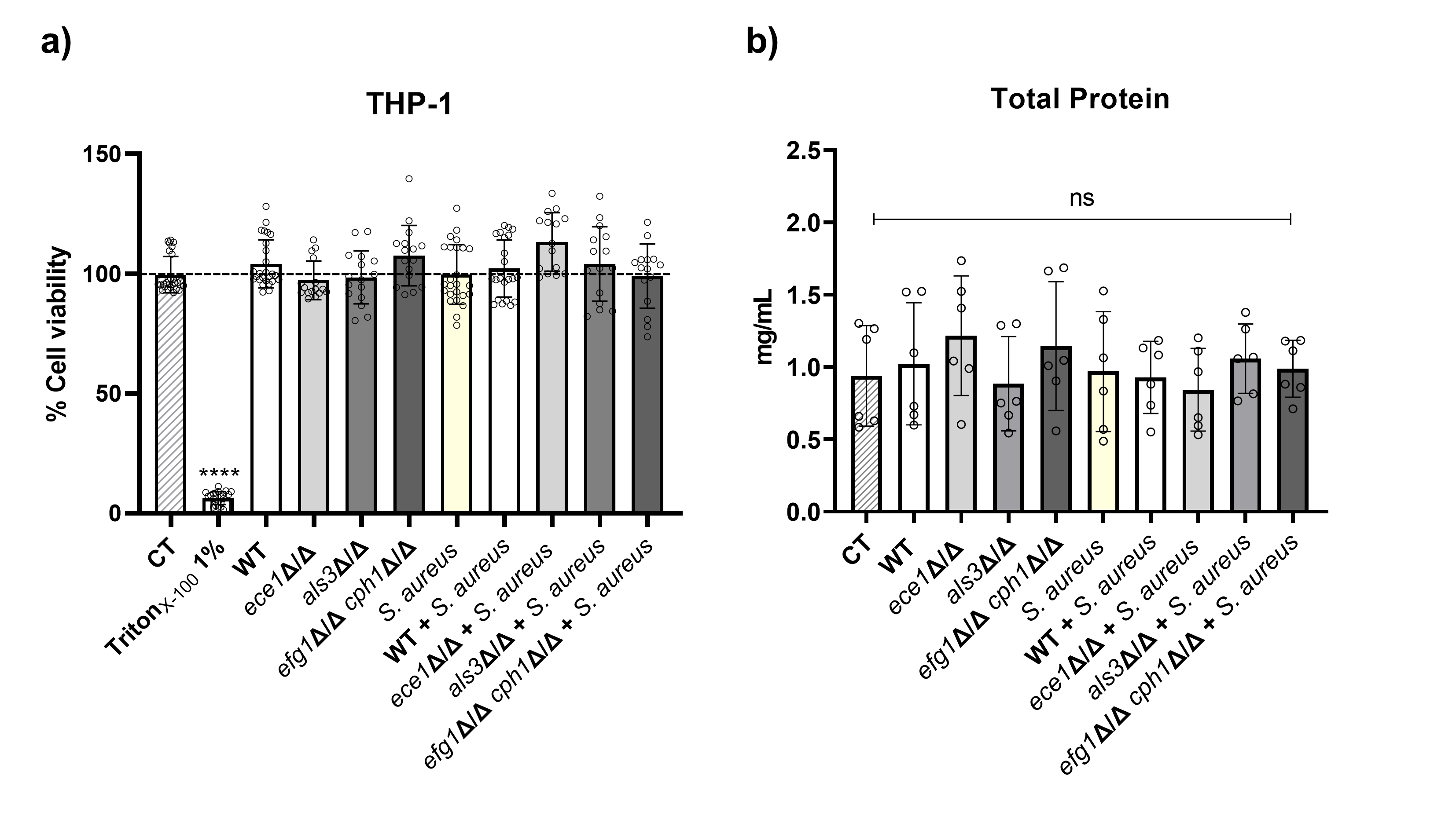

Supplement: Supplementary file 2 — Supplementary file2 (TIF 17992 KB) [file 430_2026_880_MOESM2_ESM.tif]
